# Transcriptome analysis reveals modulation of the STAT family in PEDV-infected IPEC-J2 cells

**DOI:** 10.1186/s12864-020-07306-2

**Published:** 2020-12-14

**Authors:** Zhengzheng Hu, Yuchen Li, Heng Du, Junxiao Ren, Xianrui Zheng, Kejian Wei, Jianfeng Liu

**Affiliations:** 1 National Engineering Laboratory for Animal Breeding and MOA Key Laboratory of Animal Genetics and Breeding, College of Animal Science and Technology, China Agricultural University, Beijing, China; 2 Shenzhen Kingsino Technology Co., Ltd., Shenzhen, China

**Keywords:** PEDV, IPEC-J2, RNA-seq, STAT family

## Abstract

**Background:**

Porcine epidemic diarrhea virus (PEDV) is a causative agent of serious viral enteric disease in suckling pigs. Such diseases cause considerable economic losses in the global swine industry. Enhancing our knowledge of PEDV-induced transcriptomic responses in host cells is imperative to understanding the molecular mechanisms involved in the immune response. Here, we analyzed the transcriptomic profile of intestinal porcine epithelial cell line J2 (IPEC-J2) after infection with a classical strain of PEDV to explore the host response.

**Results:**

In total, 854 genes were significantly differentially expressed after PEDV infection, including 716 upregulated and 138 downregulated genes. Functional annotation analysis revealed that the differentially expressed genes were mainly enriched in the influenza A, TNF signaling, inflammatory response, cytokine receptor interaction, and other immune-related pathways. Next, the putative promoter regions of the 854 differentially expressed genes were examined for the presence of transcription factor binding sites using the MEME tool. As a result, 504 sequences (59.02%) were identified as possessing at least one binding site of signal transducer and activator of transcription (STAT), and five STAT transcription factors were significantly induced by PEDV infection. Furthermore, we revealed the regulatory network induced by STAT members in the process of PEDV infection.

**Conclusion:**

Our transcriptomic analysis described the host genetic response to PEDV infection in detail in IPEC-J2 cells, and suggested that STAT transcription factors may serve as key regulators in the response to PEDV infection. These results further our understanding of the pathogenesis of PEDV.

**Supplementary Information:**

The online version contains supplementary material available at 10.1186/s12864-020-07306-2.

## Background

Porcine epidemic diarrhea (PED), caused by the porcine epidemic diarrhea virus (PEDV), is one of the most severe and globally widespread infectious diseases affecting swine of all ages. PED has resulted in significant economic losses to the pig industry over the past three decades. It was first observed in Europe in 1971 [1]. Outbreaks occurred in Asian countries in 1982 and since then, PED has had a growing economic impact on the Asian swine industry [2]. In 2013, PEDV was first reported in the US and spread rapidly nationwide [3]. PED is characterized by severe diarrhea, vomiting, dehydration, and a mortality rate of up to 90% in suckling piglets [4].

Domestic pig farms in China have been using the epidemic diarrhea inactivated vaccine (KPEDV-9) since 2011. A more common method of immune prevention is to use infected sow feces and the intestines of infected piglets during pregnancy. The collected feces and intestines are artificially mixed and fed back (i.e., feedback method). However, the poor external biosecurity of pig farms makes the effectiveness of these preventive methods questionable because PED often occurs on farms that use vaccination or feedback methods. Therefore, research is now focused on alternative genetic-based methods, which first involves identifying specific host genes responsible for resistance to PED.

PEDV mainly infects and replicates in the villous enterocytes of the small intestine (duodenum, jejunum, and ileum) [5–7], which leads to villous atrophy and vacuolation, as well as a marked reduction in enzymatic activity. These changes interrupt the digestion and absorption of nutrients and electrolytes, leading to malabsorptive watery diarrhea in piglets [8]. The mechanism that leads to more serious disease and death following PEDV infection remains to be clearly defined [9]. In particular, receptor recognition of PEDV is still a subject of controversy. Over the last 10 years, similar to other coronaviruses, a number of studies have suggested that PEDV uses porcine aminopeptidase N (pAPN) as a cellular receptor [10]. Experiments showed biochemical interactions between PEDV S1 and APN expressed on the cell surface [11]. It can increase the susceptibility of these cells to PEDV infection when pAPN is expressed in non- tropic cell line (i.e, swine testis cells, ST cells) [12]. However, knockout of APN expression in PEDV-susceptible porcine and human cell lines (Huh7 and HeLa) confirmed that APN is not required for PEDV infection [13].

A few immune-related pathways, including the Janus kinase (JAK)/signal transducer and activator of transcription (STAT) signaling pathway, the NF-kappa B signaling pathway, and the PI3K/Akt/mTOR signaling pathway have been reported to play a role in responding to PEDV infection [14]. Importantly, the JAK-STAT signaling pathway regulates the adaptive and innate mechanisms related to mucosal immunity [15], and a previous study suggested that the JAK-STAT signaling pathway is activated after PEDV infection of host cells [16]. As an important component of the JAK-STAT signaling pathway, STAT proteins are reported to be activated in response to immunomodulation [17–19]. After activation, STAT proteins dimerize, translocate to the nucleus, and bind to the promoters of specific target genes, resulting in the regulation of expression of the target genes. To date, seven mammalian members of the STAT family have been identified, and have been suggested to play different physiological and biological roles.

Viral infection triggers changes in host gene expression patterns. In this study, to investigate the host response patterns to PEDV infection and to identify the key regulators involved in PEDV pathogenesis, the RNA-seq technique was applied to monitor global transcriptomic changes in the intestinal porcine epithelial cell line J2 (IPEC-J2). We report how signaling pathways respond to PEDV infection and suggest that STAT proteins act as key regulators when the host is under PEDV attack. Our findings offer insight into important host-dependent factors responsible for PEDV pathogenesis in vitro.

## Results

### Response of IPEC-J2 cells to PEDV strain CV777 infection

**Fig. 1:**
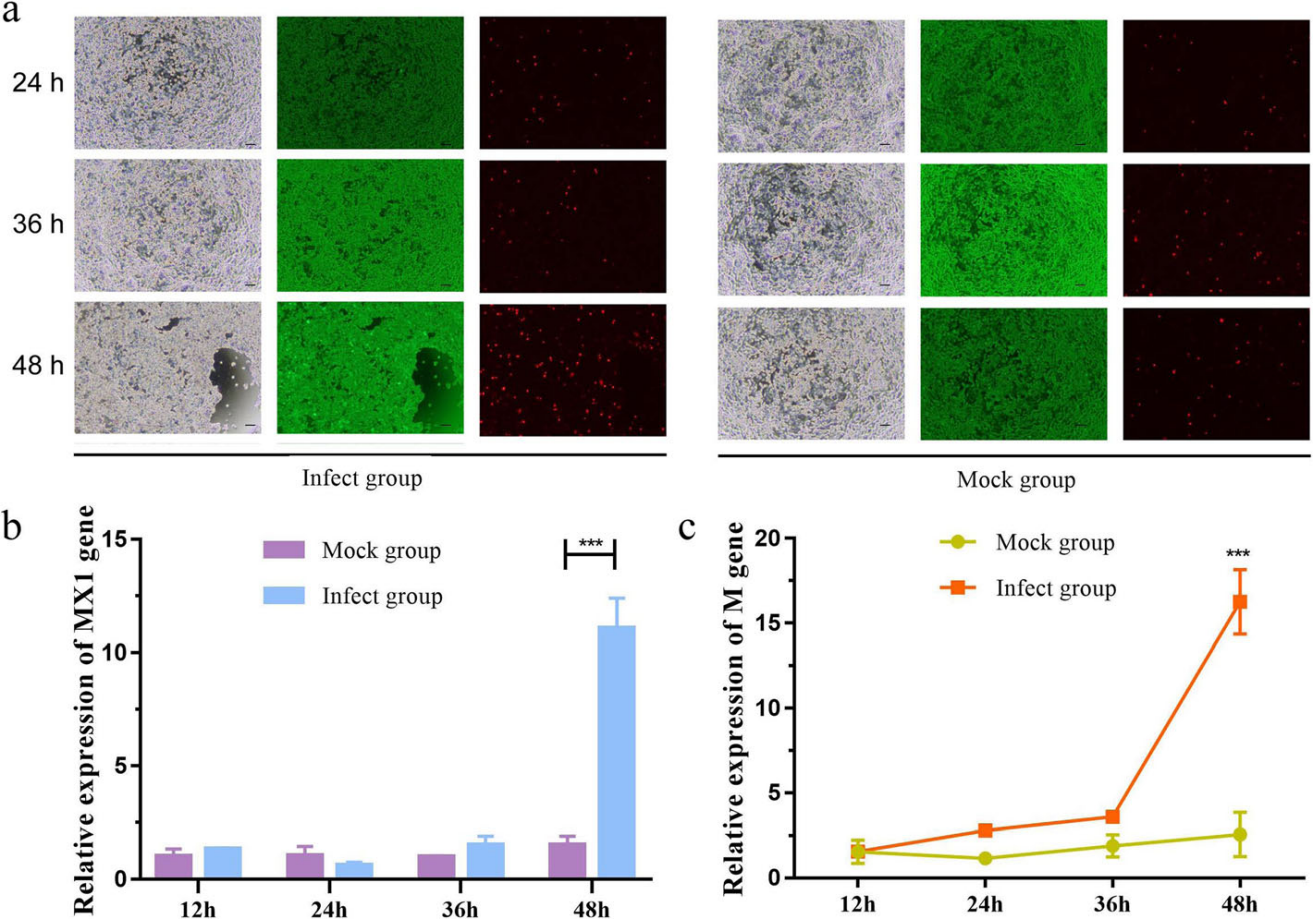
PEDV infection in IPEC-J2 cells. **a** Fluorescence-based cytotoxicity assay (using the LIVE/DEAD Viability/Cytotoxicity kit) of IPEC-J2 cells treated with PEDV strain CV777 at different time points. The green color indicates live cells stained by Calcein-AM and the red color indicates dead cells stained by propidium iodide. The green color was predominant in all viability assays, with only a few red (dead) cells appearing randomly. A significantly higher percentage of apoptotic cells (live (green)/dead (red)) are observed in the infected groups than in the controls (magnification, ×40; bars, 200 μm). **b** The mRNA expression level of *MX1* gene in PEDV-infected IPEC-J2 cells as measured by qRT-PCR at different time points. **c** The mRNA expression level of *M* gene in PEDV-infected IPEC-J2 cells as measured by qRT-PCR at different time points. Data derive from three independent experiments and were analyzed by one-way ANOVA. Data are presented as the mean ± SD (n = 6). *** represents *p* < 0.001

To detect the effects of PEDV strain CV777 on the growth phenotype of cells, healthy IPEC-J2 cells were infected with CV777 at a multiplicity of infection (MOI) of 1.0. As shown in Fig. 1a, the apoptosis rate increased with the increase in infection time. To further evaluate the response of IPEC-J2 cells to CV777 infection, we then detected the expression of the MX dynamin-like GTPase 1 (*MX1*) gene, a classic antiviral gene [20]. The results showed that MX1 mRNA expression was significantly increased after 48 h of CV777 infection (Fig. 1b). Next, the relative transcription level of the CV777 membrane protein (*M*) gene was also calculated. As shown in Fig. 1c, a significant increase in the expression level of *M* gene was detected 48 h after inoculation. These results suggested that it takes 48 h for CV777 to replicate extensively in IPEC-J2 cells and for IPEC-J2 cells to produce strong antiviral responses.

### RNA-seq analysis

**Fig. 2:**
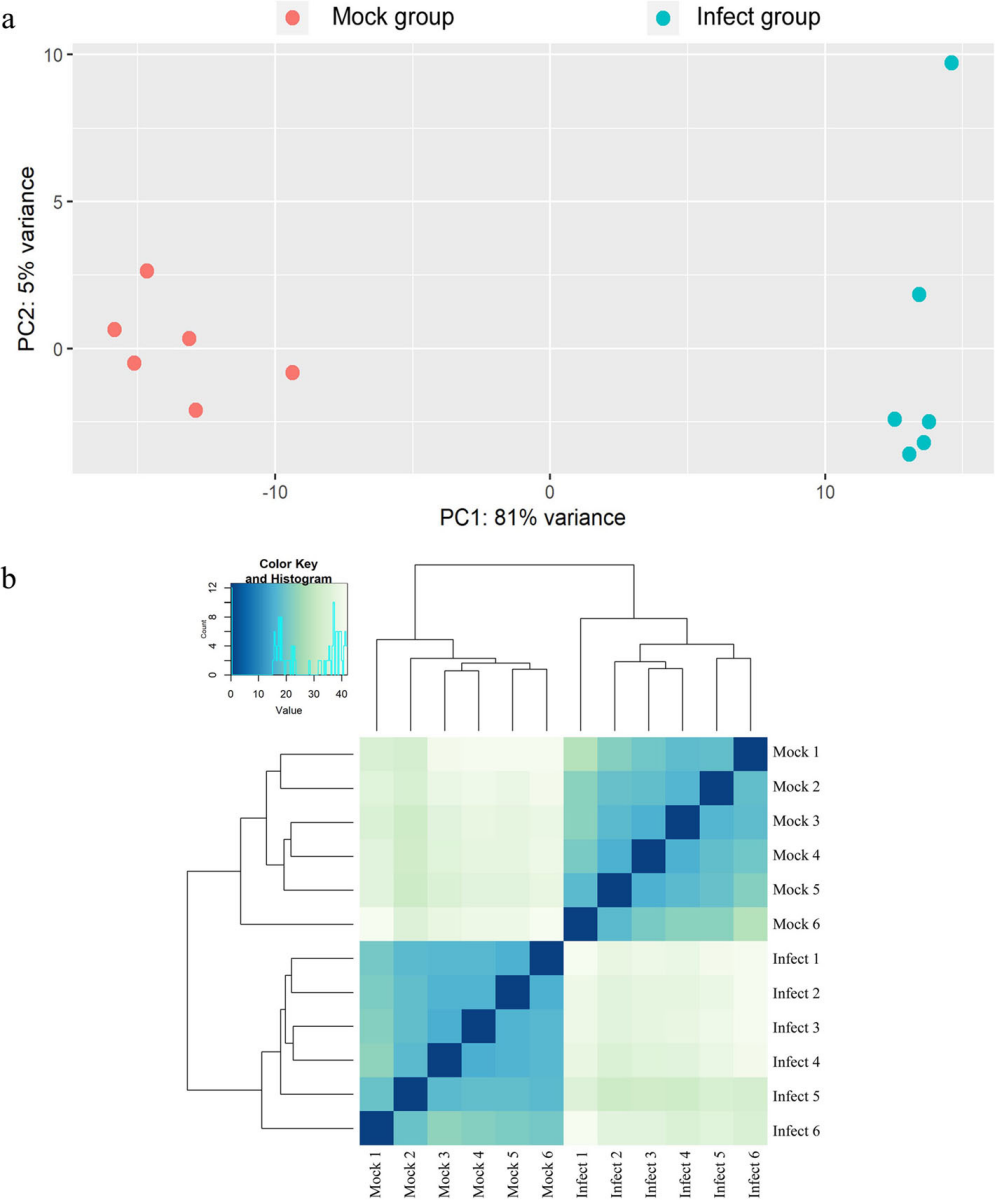
Analysis of sample correlation. **a** PCA analysis based on the gene expression profile of each sample. **b** Heatmap shows the Pearson’s correlation of gene expression levels between samples

To determine how global gene expression is differentially influenced by PEDV infection, RNA-seq was performed to compare gene expression between IPEC-J2 cells infected with PEDV for 48 h and mock-infected control cells. To avoid sample bias, six replicates for each group were collected and used for the construction of the RNA-seq libraries. The 12 libraries were sequenced on an Illumina HiSeq 2500 sequencer, and a total of 23.0–29.9 million paired raw reads were generated for each library. After removing low-quality reads and reads with adaptor sequences, we obtained approximately 22.9–29.9 million paired clean reads. Subsequently, the clean reads were aligned to the pig genome (*Sus scrofa 11.1*) and all samples had mapping ratios within the range of 91.86–92.99% (Table 1). Principal component analysis showed distinct separation between groups and confirmed the reproducibility of our biological samples (Fig. 2a). A sample correlation matrix based on gene expression levels further emphasized the high reproducibility and reliability of our experimental samples (Fig. 2b).

**Table 1:** Descriptive summary of data generated by RNA-Seq

Sample ID	Raw reads	Clean reads	Clean ratio	Total reads for alignment (QC-passed reads + QC-failed reads)	Mapped reads (total)	Mapped ratio (total)	Mapped reads (paired)	Mapping ratio (paired)
Infect 1	46,065,300	45,922,764	0.996905784	49,636,717	45,797,615	92.27%	38,974,528	84.87%
Infect 2	55,562,182	55,406,236	0.997193307	59,954,026	55,360,811	92.34%	47,118,286	85.04%
Infect 3	50,507,214	50,357,430	0.997034404	54,364,730	50,035,130	92.04%	42,426,394	84.25%
Infect 4	52,906,558	52,752,936	0.997096352	57,115,291	52,716,642	92.30%	45,013,566	85.33%
Infect 5	59,979,834	59,803,794	0.997065014	64,780,302	59,797,269	92.31%	51,204,340	85.62%
Infect 6	52,050,326	51,879,500	0.996718061	55,819,070	51,276,842	91.86%	43,823,066	84.47%
Mock 1	49,060,712	48,908,052	0.996888345	53,021,565	49,094,574	92.59%	41,888,032	85.65%
Mock 2	50,977,484	50,835,088	0.997206688	55,172,358	51,304,563	92.99%	43,812,718	86.19%
Mock 3	46,718,068	46,578,322	0.997008738	50,307,889	46,715,213	92.86%	40,165,022	86.23%
Mock 4	52,353,214	52,206,814	0.99720361	56,319,798	52,293,923	92.85%	44,864,730	85.94%
Mock 5	47,101,820	46,967,422	0.99714665	50,670,957	47,005,003	92.77%	40,382,898	85.98%
Mock 6	48,607,592	48,462,936	0.997024004	52,119,972	48,147,901	92.38%	41,313,114	85.25%

*Mapped ratio (total)* Mapped reads (total)/Total reads for alignment (QC-passed reads + QC-failed reads)

*Mapping ratio (paired)* Mapped reads (paired)/Clean reads

### Identification of the host gene response to PEDV infection

**Fig. 3:**
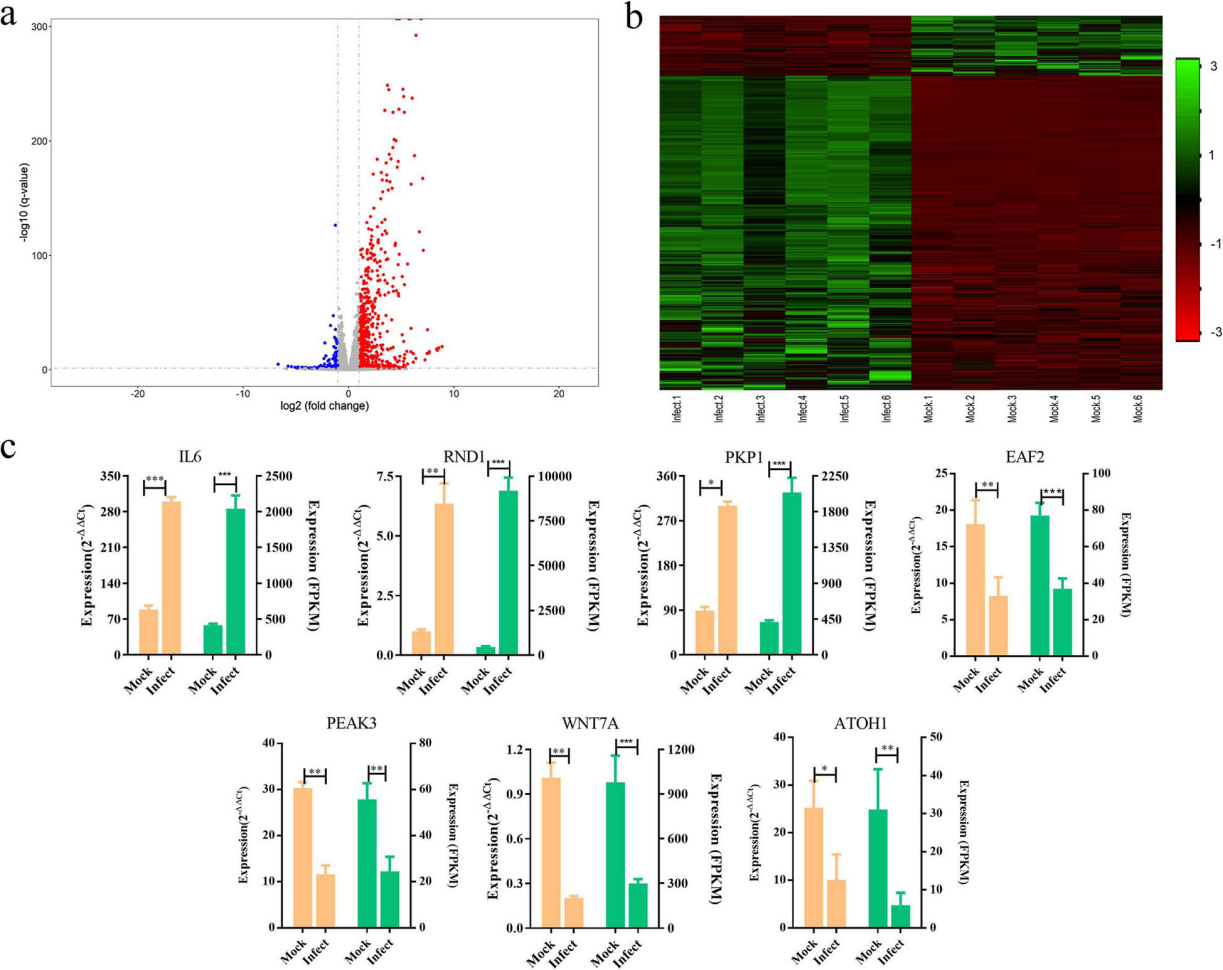
Identification of differentially expressed genes. **a** Volcano plots of differentially expressed genes between the mock-infected and PEDV-infected groups. The red and green dots represent upregulated and downregulated genes, respectively, in the PEDV-infected group compared with the mock-infected group. **b** Heatmap showing the expression levels of the differentially expressed genes. Columns represent individual samples and rows indicate genes with significant expression differences between the two groups. **c** qRT-PCR validation of the differentially expressed genes. The left axis represents gene expression levels as determined by qRT-PCR, and the right axis represents the expression levels determined by RNA-seq in FPKM units. mRNA expression levels were normalized to the mRNA levels of the pig *ACTB* gene. Graphed data represent the mean ± SD, *n* = 6. * represents *p* < 0.05, ** represents *p* < 0.01, *** represents *p* < 0.001

A total of 18,573 pig genes was found to be expressed both in mock-infected cells (*n* = 6) and PEDV-infected cells (n = 6). On comparison, a total of 716 significantly upregulated and 138 significantly downregulated genes were identified in PEDV-infected cells compared with control cells (|log_2_ fold change| ≥ 1 and FDR ≤ 0.05; Fig. 3a, b; Supplementary Table 1), including many known antiviral genes, such as interferon-induced protein with tetratricopeptide repeats 1 (IFIT1) [21], MX1, myxovirus resistance 2 (MX2) [22], and tripartite motif containing 25 (TRIM25) [23]. The top five genes with the highest changes in the mRNA level were IFIT1, DExD/H-box helicase 58 (DDX58), radical S-adenosyl methionine domain containing 2 (RSAD2), MX2, and forkhead box S1 (FOXS1). To validate the quality of the RNA-seq data, five differentially expressed genes were random selected and their expression patterns were detected by qRT-PCR. The results showed that the expression patterns of the genes were consistent with the RNA-seq results (Fig. 3c), although the observed fold changes differed between the qRT-PCR and RNA-seq data, which may reflect differences in the sensitivity and specificity between qRT-PCR and high-throughput sequencing technology. Our findings suggested that the RNA-seq results were generally reliable.

### Functional analysis of the differentially expressed genes

To better understand the functions of the differentially expressed genes, GO and KEGG pathway enrichment analyses were performed. The GO enrichment analysis showed that these genes were significantly enriched in 67 GO terms (*p* < 0.01, Supplementary Table 2). Among these, 19 (28.4%) GO terms were related to the host immune response and inflammatory response (Table 2), including the innate immune response (GO: 0045087), positive regulation of T cell proliferation (GO: 0042102), and regulation of the adaptive immune response (GO: 0002819). KEGG pathway enrichment analysis indicated that the differentially expressed genes were significantly enriched in 41 KEGG pathways (*p* < 0.01, Supplementary Table 3). Among these pathways, the NF-kappa B signaling pathway, Toll-like receptor (TLR) signaling pathway, JAK-STAT signaling pathway, and the intestinal immune network for IgA production signaling pathway were associated with the innate immune response and inflammatory response (Table 3). Interestingly, almost all differentially expressed genes that clustered in these immune-related GO and KEGG pathways were found to be upregulated after CV777 inoculation. For example, interferon regulatory factor 7 (IRF7), C-C motif chemokine ligand 5 (CCL5), STAT1, and interleukin 6 (IL6), which are clustered in the innate immune response (GO: 0045087), were all upregulated after PEDV infection. In the NF-kappa B signaling pathway, all differentially expressed genes, including CD40 molecule, TRIM25, and nuclear factor kappa B subunit 1 A (NFKB1A), were found to be upregulated. These results showed that the innate immune response and inflammatory response were activated during the course of CV777 infection.

**Table 2:** The GO terms were related to host immune response and inflammatory response

Term	Count	%	*P*-Value
GO:0006954 ~ inflammatory response	33	4.5143639	2.37E-12
GO:0006955 ~ immune response	33	4.5143639	1.16E-11
GO:0050727 ~ regulation of inflammatory response	10	1.3679891	2.24E-06
GO:0045087 ~ innate immune response	20	2.7359781	4.18E-05
GO:0007259 ~ JAK-STAT cascade	6	0.8207934	1.49E-04
GO:0051897 ~ positive regulation of protein kinase B signaling	9	1.2311902	1.85E-04
GO:0050729 ~ positive regulation of inflammatory response	8	1.0943912	3.99E-04
GO:0043491 ~ protein kinase B signaling	6	0.8207934	0.0022717
GO:0042102 ~ positive regulation of T cell proliferation	7	0.9575923	0.0032181
GO:0032649 ~ regulation of interferon-gamma production	3	0.4103967	0.0046683
GO:0060337 ~ type I interferon signaling pathway	3	0.4103967	0.0046683
GO:0002819 ~ regulation of adaptive immune response	3	0.4103967	0.0046683
GO:0060333 ~ interferon-gamma-mediated signaling pathway	3	0.4103967	0.0046683
GO:0071347 ~ cellular response to interleukin-1	6	0.8207934	0.0072168
GO:0051092 ~ positive regulation of NF-kappaB transcription factor activity	9	1.2311902	0.0078558
GO:0034341 ~ response to interferon-gamma	4	0.5471956	0.0082652
GO:0002523 ~ leukocyte migration involved in inflammatory response	3	0.4103967	0.0090894
GO:0072602 ~ interleukin-4 secretion	3	0.4103967	0.0090894
GO:0071357 ~ cellular response to type I interferon	3	0.4103967	0.0090894

**Table 3:** The KEGG pathway were related to host immune response and inflammatory response

Term	Count	%	*P*-Value
ssc04668: TNF signaling pathway	22	3.0095759	1.44E-09
ssc04672: Intestinal immune network for IgA production	13	1.7783858	7.83E-08
ssc04064:NF-kappa B signaling pathway	17	2.3255814	4.29E-07
ssc04620: Toll-like receptor signaling pathway	18	2.4623803	4.97E-07
ssc04630: Jak-STAT signaling pathway	21	2.872777	7.57E-07
ssc05321: Inflammatory bowel disease (IBD)	13	1.7783858	4.62E-06
ssc04622: RIG-I-like receptor signaling pathway	12	1.6415869	7.85E-05
ssc04151: PI3K-Akt signaling pathway	22	3.0095759	0.023559

### Transcription factor prediction among differentially expressed genes

**Fig. 4:**
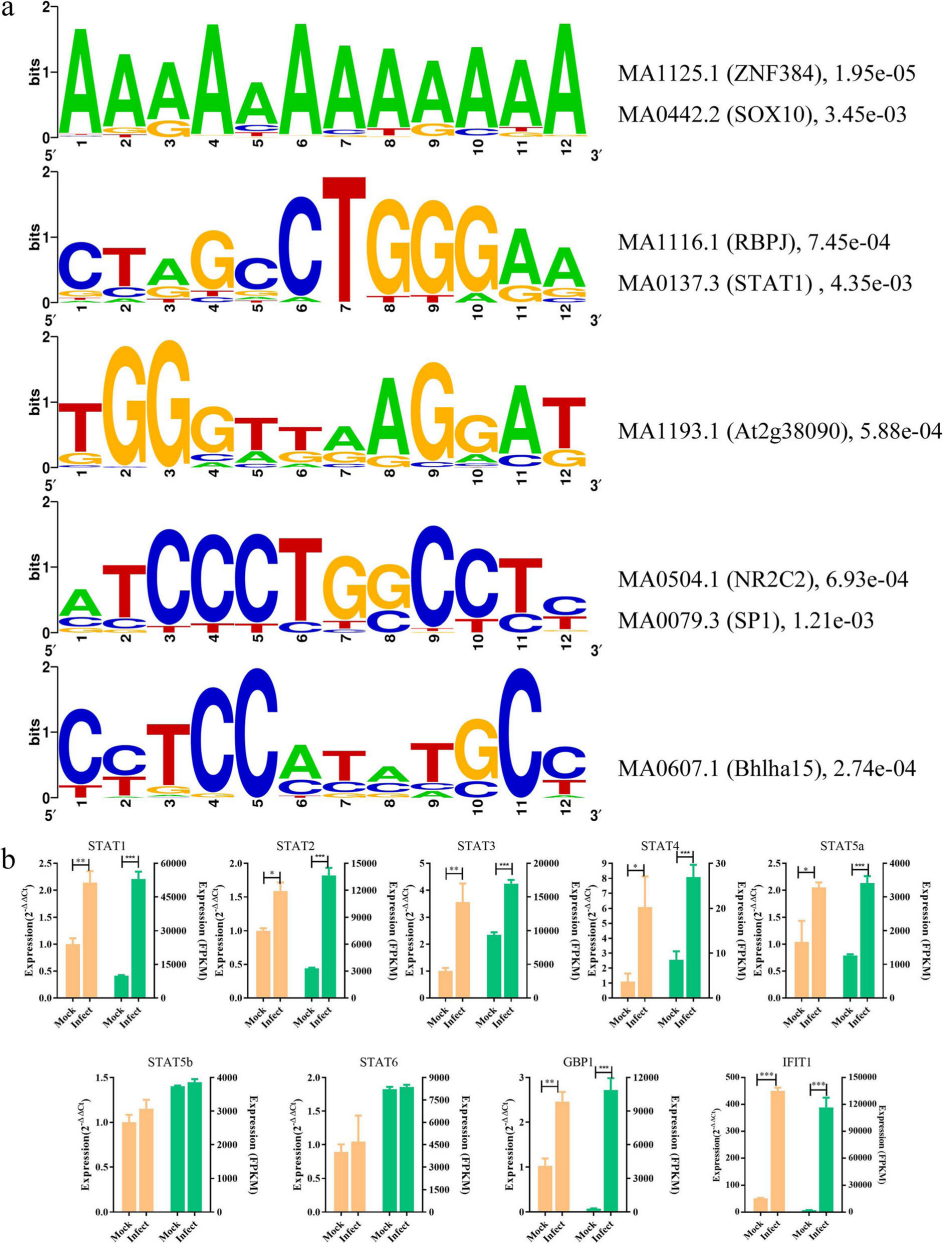
Prediction of potential transcription factors. **a** Enrichment of putative transcription factor binding motifs in the promoter regions of differentially expressed genes detected by MEME. The right panel shows the annotation of the enriched motifs as determined by MAST (ID, number, p value). **b** qRT-PCR validation of STAT expression levels and those of their known target genes. The left axis represents the gene expression levels determined by qRT-PCR, and the right axis represents the expression levels determined by RNA-seq in FPKM units. mRNA expression levels were normalized to the mRNA levels of the pig *ACTB* gene. Graphed data represent the mean ± SD, *n* = 6. * represents *p* < 0.05, ** represents *p* < 0.01, *** represents *p* < 0.001

The search for significantly overrepresented transcription factor binding sites in the promoter regions of the differentially expressed genes could be a powerful approach for finding key regulators of complex biological processes. Therefore, the putative promoter regions (2000 bp upstream of the transcription start site) of the differentially expressed genes were examined for the presence of transcription factor binding sites using the MEME tool. This analysis revealed several significantly enriched motifs (Supplementary Fig. 1), which were then annotated as the motif of transcription factors including zinc finger protein 384 (ZNF384), SRY-box transcription factor 10 (SOX10), STAT1 (a member of the STAT family), and recombination signal binding protein for immunoglobulin kappa J region (RBPJ), among others (Fig. 4a). In particular, five STAT members—STAT1, STAT2, STAT3, STAT4, and STAT5a—were identified as differentially expressed genes by RNA-seq (Fig. 4b). Subsequently, all of the STAT members and their known target genes, *GBP1* [24, 25] and *IFIT1* [26], were verified by qRT-PCR and the results were consistent with the RNA-seq data (Fig. 4b). To corroborate these findings, the Find Individual Motif Occurrences (FIMO) tool was used to identify putative STAT1 binding sites in these sequences using the count matrix motif of STAT obtained from the JASPAR database (ID: MA0137.3). As a control, another set of promoter sequences extracted from 854 randomly-selected genes were subjected to the same FIMO analysis. As a result, 504 (59.02%) promoter sequences for the differentially expressed genes were identified to have at least one STAT1 binding site. By comparison, only 112 (13.11%) of sequences among the control promoter sequences were identified as having a STAT1 binding site. The highly significant overrepresentation (*p* < 0.001, Pearson’s Chi-square test) of STAT1 transcription factors among the putative promoters of the differentially expressed genes suggested an important role for STAT factors during the PEDV infection process.

### Construction of a gene regulatory network between the STAT factors and the differentially expressed genes

**Fig. 5:**
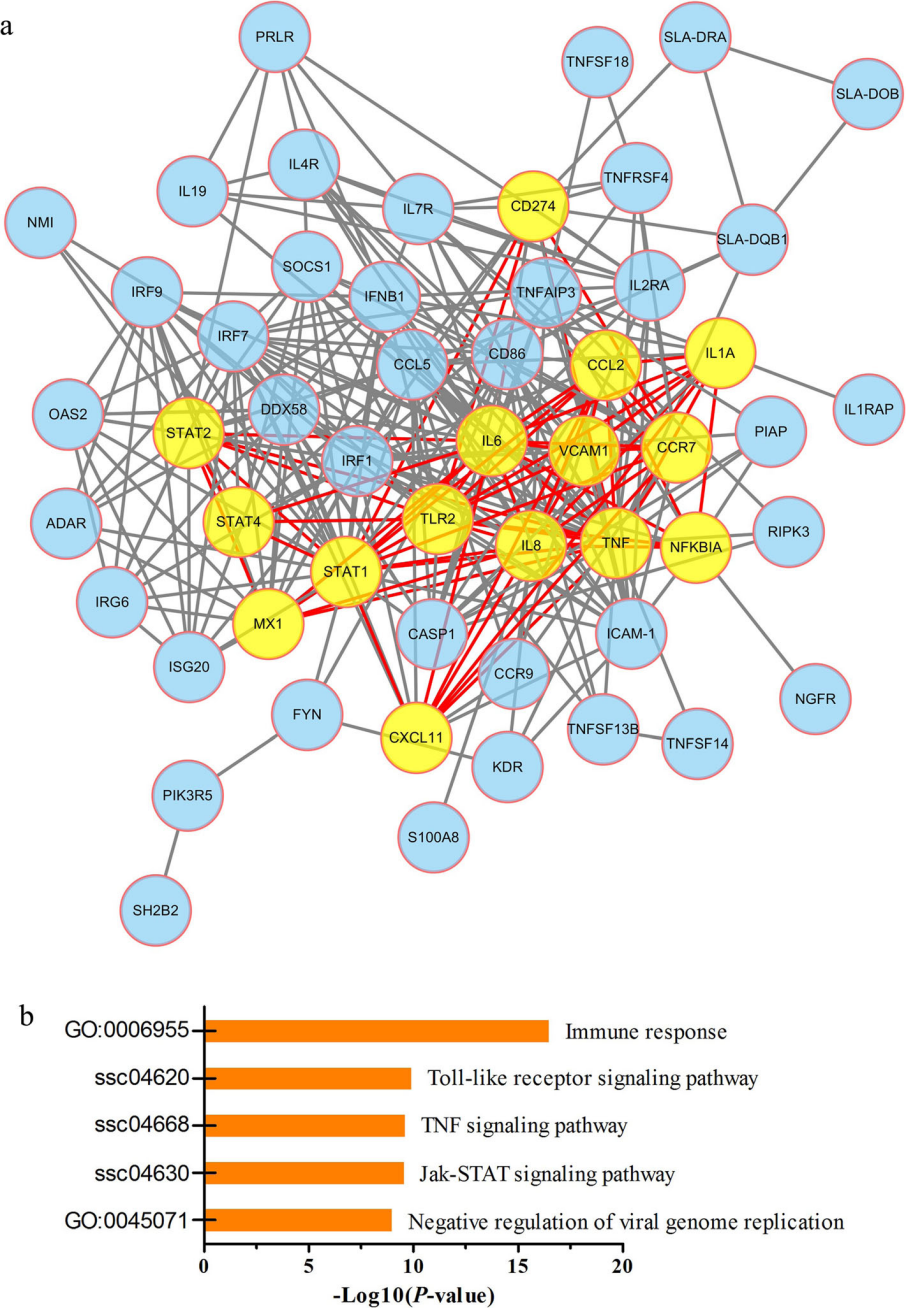
STAT transcription factors as key regulators during PEDV infection. **a** PPI network of the differentially expressed genes based on STRING analysis. The hub genes of the network are shown in yellow. **b** Top five pathways enriched in immune response-related functions. GO and KEGG analyses were conducted using a DAVID functional annotation program

On the basis of the assumption that the STAT protein family-mediated regulatory and signaling networks are representative of the infected interactome, STAT factors were used as seeds to construct a gene regulatory network. Figure 5a shows the nodes and interactions at the intersection of the network. GO and KEGG analyses showed that these genes were highly enriched in immune response-related functions (Fig. 5b). Additionally, topological analysis indicated that *IL6*, tumor necrosis factor (*TNF*), *NFKB1A*, *MX1*, and *TLR2* are the principal hub genes, and all these genes are recognized as important immune response genes. Taken together, our results further implicate STAT members as key regulators when the host is under PEDV attack.

## Discussion

PEDV is a pathogen of interest to researchers because of its significant impact on the swine industry worldwide. Many previous studies have focused on viral isolation and molecular epidemiology surveys [27, 28]; however, it is also necessary to understand the molecular mechanisms involved in the host response to PEDV infection. Our study, which involved transcriptome analyses, revealed 854 significantly differentially expressed genes in the host. These differentially expressed genes were mainly enriched in the influenza A, TNF signaling pathway, inflammatory response, and other immune-related pathways. In particular, five members of the STAT family were significantly upregulated in PEDV-infected cells compared with mock-infected cells. STAT factors are considered to be downstream mediators of cytokine signaling. STAT proteins are capable of rapid and direct transduction of signals from the cell membrane to the nucleus, and they combine the functions of transduction agents and inducers of transcription. In the latent state, STAT proteins are inactive as monomers or unphosphorylated dimers, which are localized in the cytoplasm of unstimulated target cells [29–32]. As a non-negligible component of the JAK-STAT signaling pathway, STAT factors play an indispensable role in innate immunity to viral infection. Each member of the STAT family can be activated by multiple cytokines and their associated JAKs [33]. STAT1 plays a role in many important cytokine induction pathways and can upregulate many pro-inflammatory cytokines, whereas STAT2 is a co-factor in the type I IFN signaling pathway. Previous studies have shown that PEDV infection inhibits type I IFN induction [34]. Guo et al. demonstrated that PEDV infection promotes the degradation and interrupts the activation of STAT1 without inhibiting STAT1 transcription, while STAT2 remains uncleaved [35]. As the most important activator of STAT3, IL6 can directly act on target cells to affect STAT3 expression [36]. Yang et al. demonstrated that the direct interaction between PEDV S protein and epidermal growth factor receptor (EGFR) induces EGFR activation, and thus increases PEDV infection. They further demonstrated that activation of EGFR contributes to the enhancement and promotion of PEDV replication via JAK-STAT3 signaling pathways [37]. Our transcriptome data are consistent with previous studies. However, no significant change was observed in STAT5b and STAT6 expression after PEDV infection. Curiously, STAT5a and STAT5b, a pair of homologous genes that exhibit high similarity (~ 94%) in their coding sequences, showed different expression patterns after PEDV infection. STAT5a and STAT5b have multiple functions, including cell proliferation and differentiation [38], immunoregulation [39], a drug response, and metastasis [29]. STAT5a and STAT5b often perform similar functions in mediating regulatory signaling. However, recent studies have shown that STAT5a and STAT5b can also have distinct roles in regulating gene expression [40]. Lamba et al. reported the distinct and potentially opposing roles of STAT5a and STAT5b in the regulation of hepatic drug response genes [41]. STAT5a is expressed at a much lower level in the liver than STAT5b [42]. Jennifer et al. reported the differentially regulated expression of FOXP3 and IL-2R in STAT5b knockdown human primary T cells and the downregulated expression of Bcl-X only in STAT5a knockdown human primary T cells [43]. In the present study, our findings revealed for the first time that STAT5a, but not STAT5b, regulated the expression of key factors involved in the immune response after virus infection.

Previous studies provided transcriptomic and proteomic data for PEDV-infected Vero cells that offered the first valuable insight to better understand the host response to PEDV infection [16, 44, 45]. However, these reports contained some contradictory results. For example, nearly all of the detected IRFs were reported to be downregulated after PEDV infection in Vero E6 cells [44], whereas our results showed that IRF1, FIRF2, IRF7, and IRF9 were significantly upregulated after PEDV infection in IPEC-J2 cells, and other detected IRF members were not significantly changed. These conflicting results are most likely due to the different cell lines or the different detection times after infection used between studies. With this in mind, we believe that more integrated and detailed studies of time-resolved or even single-cell-resolved gene expression changes will aid in understanding the host gene response to PEDV infection. A recent study performed global mapping of H3K4 trimethylation and transcriptomic analyses in the PEDV-infected jejunum of piglets compared with healthy piglets, using chromatin immunoprecipitation sequencing and RNA-seq, and provided novel insights into our understanding of the host response to PEDV infection [14]. RSAD2, an important antiviral gene [46], has attracted extensive attention recently. Jiang et al. confirmed that RSAD2 is necessary for mouse dendritic cell (mDC) maturation via the IRF7-mediated signaling pathway, and mDCs were shown to lose their antitumor efficacy in a RSAD2-knockdown lung metastasis mouse model [47]. IRFs are involved in antiviral defense and immune regulation [48, 49]. In particular, IRF3 and IRF7 are direct transducers of virus-mediated signaling in the induction of type I IFN [50]. Our results showed that the mRNA expression of RASD2 and IRF7 was significantly upregulated, while the mRNA expression of IRF3 did not change after PEDV infection. This result suggested that IRF7, but not IRF3, might be regulated by RSAD2 to initiate the antiviral response.

Viruses have the ability to manipulate host cell signal transduction pathways to promote their own survival [51]. In our study, significantly enriched pathways in response to PEDV infection included the protein kinase B signaling (Akt) pathway, which modulates the cell cycle, apoptosis, and differentiation. It is well established that the regulation of Akt activity is mainly dependent upon PI3K activity. Unexpectedly, phosphoinositide-3-kinase adaptor protein 1 (PIK3AP1), a member of the vital PIKs, was significantly upregulated during PEDV infection at the transcriptional level. The mechanism by which PEDV manipulates the PIK/Akt pathway to achieve maximum virus replication is worth studying.

## Conclusion

In this study, changes in a series of immune and inflammatory response-related genes in PEDV CV777-infected IPEC-J2 were observed by RNA-seq analysis. Among the total gene set, 59.02% of fragments were identified as possessing at least one putative binding site for STAT transcription factors. Moreover, a STAT member-induced regulatory network, including 53 genes that were differentially expressed during the process of PEDV infection, was constructed. These results will be useful for exploring and further understanding host responses to PEDV infection in pigs.

## Methods

### Cells and virus

IPEC-J2 cells were grown in antibiotic-free Dulbecco’s modified Eagle’s medium (DMEM)/F12 (Gibco, Rockville, MD, USA) supplemented with 5% fetal bovine serum (Gibco), insulin (5 μg/mL, Gibco), transferrin (5 μg/mL, Gibco), selenium (5 ng/mL, Gibco), and epidermal growth factor (5 ng/mL, Sigma-Aldrich, St. Louis., MO, USA). Cells were maintained in 100 mL flasks at 37 °C in an atmosphere of 5% CO_2_. The medium was replaced every 2 days.

CV777 strain of porcine epidemic diarrhea virus was provided by the College of Animal Medicine of China Agricultural University. The IPEC-J2 line was initially obtained from the American Type Culture Collection (ATCC, Manassas, VA, USA).

### PEDV infection of IPEC-J2 cells

Culture medium was removed when the monolayer of IPEC-J2 cells had grown to 80% confluence in 10 cm^2^ cell culture dishes. After washing gently with PBS, cells were incubated in serum-free DMEM and inoculated with CV777 at a multiplicity of infection of 1. The serum-free DMEM was replaced with culture medium after 1 h of adsorption. Cells were harvested after another 12, 24, 36, and 48 h of culture, and were infected with PEDV. Cells incubated in serum-free DMEM without inoculation of CV777 served as the mock-infected control group.

### Cell apoptosis detection

Cell apoptosis was detected using the TransDetect Cell LIVE/DEAD Viability/Cytotoxicity Detection Kit (Trans, Beijing, China) according to the manufacturer’s protocol. Briefly, culture medium was removed, and cells were washed once with PBS. Cells were then incubated in a mixture of 1 μL of 1000× Calcein-AM solution, 1 μL of 1000× propidium iodide (PI) working solution, and 500 μL of PBS for 15 min at 37 °C in the dark. Cells were then immediately observed using a fluorescence microscope (Nikon, Tokyo, Japan). Live cells were marked with green fluorescence and apoptotic cells were marked with red fluorescence.

### RNA-seq

RNA-seq was performed to compare gene expression profiles between PEDV-infected IPEC-J2 cells and uninfected IPEC-J2 cells over a 48 h time period. Six biological repeats for each group were performed to prepare the RNA-seq library. The RNeasy mini kit (Qiagen, Hamburg, Germany) was used to isolate total RNA from the 12 samples according to the manufacturer’s instructions. Total RNA was purified using the RNeasy micro kit (Qiagen) and RNase-Free DNase Set (Qiagen). The RNA concentration was determined using the NanoDrop ND-2000 spectrophotometer (Thermo Fisher Scientific Inc., USA). RNA integrity was checked using the RNA Integrity Number (RIN) generated by an Agilent Bioanalyzer 2100. RNA samples with RIN ≥ 7 were used for library construction. Sequencing libraries were constructed using the TruSeq®RNA Sample Preparation Kit v2 (Illumina, San Diego, CA, USA) following the manufacturer’s protocol. Briefly, RNA was initially purified using poly-T oligo-attached magnetic beads. The purified RNA was fragmented into 100–400 bp small pieces using divalent cations at 94 °C for 8 min. The double-stranded cDNA was synthesized by priming with random hexamers. A single nucleotide A (adenine) was added (A-tailing) to the 3′ end of the end-repaired DNA fragments. The repaired fragments were ligated to sequencing adapters. The products were purified by agarose gel electrophoresis and were used as template in PCR. The minimal number of PCR cycles (15 cycles) was used to avoid skewing the representation of the library. The final cDNA libraries were quantified using a Qubit® 2.0 Fluorometer (Life Technologies, Carlsbad, CA, USA) and validated using an Agilent Bioanalyzer 2100 (Agilent Technologies). Finally, 12 libraries were sequenced on the Illumina HiSeq 2500 sequencer (Illumina). Transcriptome sequencing was entrusted to Beijing Boao Jingdian Biological Co., Ltd.

### Sequence bioinformatics

Raw data were cleaned using BBMap (https://sourceforge.net/projects/bbmap/) to remove sequencing linkers, primer sequences, and low-quality reads. Then, the quality-controlled sequencing data were aligned to the downloaded pig reference genome using TopHat2 software [52]. The pig reference genome (*Sus scrofa 11.1*) and the corresponding annotation file were downloaded from the Ensembl database. Then, Cufflinks software was employed to quantify the fragment per kilobase of exon model per million mapped reads (FPKM) values for each gene. The Cuffdiff software was used to screen the differentially expressed genes [53] . The significance threshold for the differential expression was FDR < 0.05 and a |log_2_ fold change | > 1.

### GO and KEGG pathway enrichment

Functional enrichment of the gene module was analyzed using the web-based tools in DAVID (v6.8; https://david.ncifcrf.gov/) to identify enriched GO terms and KEGG pathways. Ensembl gene IDs were submitted to the Gene Functional Classification Tool, then biological process, cellular component, molecular function, and KEGG pathway were selected to perform the enrichment. GO terms and KEGG pathways with *p* < 0.05 were defined as significantly enriched.

### Transcription factor binding motif analysis

We interrogated enrichments of transcription factors in the promoters of differentially expressed genes using the MEME Suite (http://meme-suite.org/index.html). First, the putative promoter regions (2000 bp upstream of the transcription start site) of the differentially expressed genes were extracted using the BioMart program in the Ensembl database. Then, these promoter sequences were employed to discover enriched motifs using the MEME tool under the default parameters. The discover program was run repeatedly until the five most enriched motifs were discovered. Subsequently, the discovered motifs were compared against the JASPAR CORE database using the Tomtom tool, resulting in exact motif annotations. Additionally, the FIMO tool was employed to scan the set of promoter sequences for individual matches to specific motifs obtained from the JASPAR CORE database. FIMO analysis was performed with stringent criteria including a *p* value <5E˗4 and a maximum of two mismatched residues.

### Construction of a gene regulatory network

The STRING (Search Tool for the Retrieval of Interacting Genes, http://string-db.org/, version 11.0) database, which provides both predicted and experimental interaction information for proteins [54], was used to construct the gene regulatory network. Interactions with a combined score > 0.4 were considered statistically significant. We then exported the results to Cytoscape v3.7.2 to visualize the gene regulatory network. In the network, each node represents a protein and each edge represents an interaction between two proteins.

### Quantitative real-time PCR

One microgram of RNA from each sample was used to synthesize cDNA using the PrimeScript™ RT reagent kit with gDNA Eraser (TaKaRa Biotech, Dalian, China) according to the manufacturer’s instructions. qRT-PCR analysis was carried out using SYBR Green master mix (Roche, USA). Each reaction contained 5 μL of SYBR Green PCR Master Mix (TaKaRa), 0.5 μL of forward primer, 0.5 μL of reverse primer, 0.5 μL of cDNA, and 3.5 μL of RNase-free water. The qRT-PCR program was as follows: 95 °C for 5 min; followed by 40 PCR cycles at 95 °C for 30 s, 60 °C for 30 s, and 72 °C for 20 s; and then a further 10-min extension at 72 °C. Each individual sample for qRT-PCR was run in triplicate. The gene expression levels were measured using the 2^˗△△Ct^ method. The pig *ACTB* gene, a housekeeping gene, was used as an internal control. All primers were designed using the NCBI Primers-BLAST online programs (https://www.ncbi.nlm.nih.gov/tools/primer-blast/) and their sequences are listed in Supplementary Table 4.

### Statistical analysis

Statistical analyses of the qRT-PCR results were carried out using SPSS 20.0 (IBM Corp., Armonk, NY, USA). The gene expression levels are presented as the mean ± SD and differences between groups were evaluated using one-way ANOVA (Dunnett’s t-test) and a two-tailed Student’s t-test.

## Supplementary Information

**Additional file 1: Supplementary Figure 1.** Motifs discovered using the MEME tool.

**Additional file 2: Supplementary Table 1.** Information on each significantly differentially expressed gene (*p* < 0.05) identified in PEDV-infected cells.

**Additional file 3: Supplementary Table 2.** GO terms significantly enriched among the differentially expressed genes in PEDV-infected cells.

**Additional file 4: Supplementary Table 3.** KEGG pathways significantly enriched among the differentially expressed genes in PEDV-infected cells.

**Additional file 5: Supplementary Table 4.** List of qRT-PCR primers.

## Data Availability

The datasets generated during the current study are available in the NCBI Sequence Read Archive under the accession number PRJNA599091.

## Abbreviations

PED: Porcine epidemic diarrhea
PEDV: Porcine epidemic diarrhea virus
IPEC-J2: Intestinal porcine epithelial cell lines J2
STAT: Signal transducer and activator of transcription
JAK: Janus kinase
pAPN: Porcine aminopeptidase N
MX1: MX dynamin like GTPase 1
IFIT1: Interferon-induced protein with tetratricopeptide repeats 1
MX2: Myxovirus resistance 2
TRIM25: Tripartite motif containing 25
DDX58: DExD/H-box helicase 58
RSAD2: Radical S-adenosyl methionine domain containing 2
FOXS1: Forkhead box S1
IRF7: Interferon regulatory factor 7
CCL5: C-C motif chemokine ligand 5
IL6: Interleukin 6
NFKB1A: Nuclear factor kappa B subunit 1 A
ZNF384: Zinc finger protein 384
SOX10: SRY-box transcription factor 10
RBPJ: Recombination signal binding protein for immunoglobulin kappa J region
FIMO: Find Individual Motif Occurences
EGFR: Epidermal growth factor receptor
