# Supplementary figures and images for: Transcriptome analysis reveals modulation of the STAT family in PEDV-infected IPEC-J2 cells
Source: BMC Genomics. 2020 Dec 14;21:891. doi: 10.1186/s12864-020-07306-2 (PMC7734901; doi:10.1186/s12864-020-07306-2)

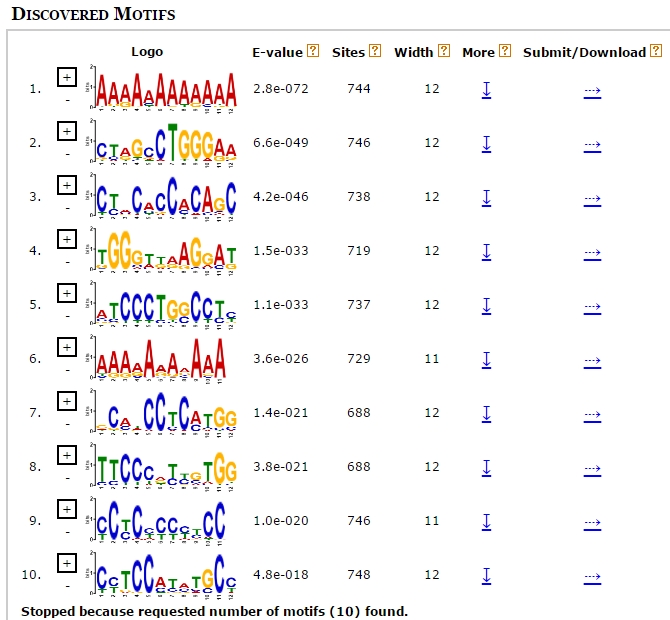

Supplement: Supplementary file 1 — Additional file 1: Supplementary Figure 1. Motifs discovered using the MEME tool. [file 12864_2020_7306_MOESM1_ESM.jpg]
